# An interview study to explore the perceived usefulness of in-house training programs in tourism and hospitality education

**DOI:** 10.1016/j.heliyon.2022.e12547

**Published:** 2022-12-22

**Authors:** Kevin Fuchs

**Affiliations:** Faculty of Hospitality and Tourism, Prince of Songkla University, Thailand

**Keywords:** Higher education, COVID-19, Competency development, Tourism and hospitality education, Undergraduate, Internships

## Abstract

COVID-19 has accelerated the adoption of online teaching and learning modes. It has also abruptly changed the traditional teaching and learning methods that originally relied on physical attendance, including student internships. In-house training programs were launched by the university to offer students an alternative to their regular internships. The study is designed to investigate how university students in tourism and hospitality education perceive the usefulness of their in-house training in absence of industry placements. An exploratory methodological approach was adopted by conducting 25 semi-structured in-depth interviews with student interns to understand their experiences during the in-house training. The empirical findings revealed that the in-house program, in its current state, is not to be considered a permanent alternative for tourism and hospitality undergraduate students. The article concludes by presenting implications for educators, policymakers, and education researchers derived from the academic and practical discussions based on the findings.

## 1 Introduction

Internships are supervised training opportunities for students that supplement academic learning in the classroom with real-world experience (Hernandez et al., 2014). The literature has identified several advantages for interns. Job-related benefits, career-related benefits, and networking/job market advantages are the primary types (Maertz et al., 2014). Furthermore, internships can be an effective technique for bridging the gap between theory and practice and improving graduate employability (Patacsil and Tablatin, 2017). Kroon and Franco (2021) validate that internships offer overall good benefits and confirm that they are a win-win situation for the three primary stakeholders: students, employers, and higher education institutions. Internships broaden students' understanding of a multicultural workplace, develop collaboration with the hotel and tourism sector, and give valuable experience for all parties involved: industry, university, and students (Seyitoğlu, 2019). Cooperation between universities and industry is critical to ensuring that students understand the role and significance of internships (Tsai et al., 2017).

It is also critical for educational institutions to design internship programs that meet the needs of both educators and industry (Plewa et al., 2015). Professional internships are an important tool for assessing students' job market performance and determining whether the training they have received meets market demands (Spanjaard et al., 2018). As a result, placements provide the ideal environment for students to develop and evaluate the professional competencies that they must acquire. Internships, as stated by Plewa et al. (2015) and Seyitoğlu (2019), have been recognized as an effective tool for improving graduates' employability competencies by industry, universities, and students (Plewa et al., 2015; Seyitoğlu, 2019; Kroon and Franco, 2021). According to the literature, internships are an important part of university studies because they help to improve competencies in areas such as communication, problem-solving, teamwork, management, planning, and decision-making (Ritter et al., 2018).

Other studies emphasize the importance of developing effective training programs (Kebritchi et al., 2017). Furthermore, companies that accept students for internships must clearly understand the activity's objectives, tasks, and requirements. If this is not the case, students will be unable to effectively develop competencies, and the internship experience will be ineffective (Jackson, 2015). COVID-19 has accelerated the adoption of online teaching and learning modes and has also abruptly changed the traditional teaching and learning methods that originally relied on physical attendance, including industry placements, i.e. student internships (Wong et al., 2021). During the global pandemic, students were largely unable to complete their industry placements and consequently, needed to postpone the implementation to a future date (Azman et al., 2020). Some universities opted for an in-house training program to avoid that their students would graduate with a significant delay (Crawford et al., 2020).

However, the perceived usefulness of in-house training as an alternative to industry placements is not clearly understood (Dhevabanchachai and Wattanacharoensil, 2017). Moreover, it can be assumed that the students’ competency development differs from a traditional internship experience (Karunaratne and Perera, 2019). Despite extensive research on internships, there are no results that report on the alternative in-house training programs that were implemented as a result. Consequently, there is a need to assess how undergraduate students perceived the usefulness of in-house training and how it affects their competency development, which is critical for future employability and career success (Chen et al., 2018). This study is exploratory and practical in nature, and hence it should be expected that it would have more practical implications, rather than theoretical ones.

It is the aim of the study to assess the students’ perceived usefulness of the alternative in-house training programs in absence of industry placements during the global pandemic. Moreover, the study aims to empirically investigate the students’ perception of their competence development during the in-house training program. To meet the aim of the study, the following research question was formulated to guide the study “*How do undergraduate students in tourism and hospitality perceive the usefulness of their in-house training in absence of an industry placement?*”.

## 2 Methodology

### 2.1 Research design

An exploratory approach (Morgan, 2013) was applied to transform a problem by investigating its complex interrelated elements to fill a gap in the existing literature about students’ experience with in-house training as an alternative to traditional internship placements. The research is being undertaken from the perspective of undergraduate students, who share their experiences with the perceived usefulness of the attended in-house training as an alternative to industry placements.

### 2.2 Participants

The participants were purposively approached by the lead researcher and asked for their participation in the study. The students were contacted in person and contact information was exchanged to agree on a suitable date and time for the interview. A total of 30 students were approached, whereas 25 confirmed their willingness to participate in the study. At the time of sampling, the majority of second and fourth-year students at the Faculty of Hospitality and Tourism, Prince of Songkla University were attending the in-house training program, if unsuccessful to find an industry placement themselves. Other sociodemographic characteristics (such as gender, year of study, age, academic major, and in-house program) of the participants are summarized in Table 1 based on the empirical survey data. The sample size of 25 can be considered sufficient (Malterud et al., 2016) and the author considers to have reached data saturation based on the definition set forth by Fusch and Ness (2015) indicating that “the ability to obtain additional new information has been attained, and when further coding is no longer feasible” (p. 1408).

**Table 1:** Socio-demographic characteristics of the participants.

Characteristic	Frequency	Percentage
**Age**		
18–19 years old	4	16%
20–21 years old	17	68%
22 years old and above	4	16%
**Gender**		
Male	11	44%
Female	14	56%
**Academic major**		
Tourism Management	16	64%
Hospitality Management	9	36%
**Year of study**		
Second-year	12	48%
Fourth-year	13	52%
**In-house program**		
Entrepreneur Prep Up	10	40%
Tour Guide Prep Up	15	60%

### 2.3 Data collection tools

Twenty-five (25) semi-structured interviews were conducted with full-time undergraduate students at the Prince of Songkla University in Phuket, Thailand. The semi-structured interviews were conducted in April 2022 by the lead investigator and were supported by a research assistant. The interviews were completed in-person off-campus within the vicinity of the university. The semi-structured interviews were guided by an eleven-item interview guide (Table 2) with appropriate probing questions for each item. The length of the interviews ranged from 20 min to 37 min with an average duration of 27 min.

**Table 2:** Interview guide for the semi-structured interviews.

No.	Question
**Opening question**	
1	What was your motivation to conduct the in-house training program instead of an industry placement?
**Self-evaluation of interns’ work performance**	
2	What was your expectation of this alternative to the internship?
3	What did you learn from the in-house training program?
4	Did you encounter any difficulties when completing the in-house training?
**Comparison between the in-house training and industry placement**	
5	Do you like the in-house training? Why and why not?
6	Comparing your expectations between an industry placement and in-house training, which is better? Why?
7	What do you think the university has done to facilitate your effectiveness when doing the in-house training?
8	What do you think that your university can do further to facilitate reaching your learning outcomes?
**Skills and competency development**	
9	Do you think that the in-house training gave you sufficient opportunities to improve your skills and competencies?
10	What skills or competencies did you improve the most during the training?
11	What skills or competencies did you improve the least during the training?

### 2.4 Data analysis

The empirical data was analyzed thematically using the methods described by Braun and Clarke (2014). The thematic analysis aims to identify common themes among the interviewed students and is a flexible qualitative analysis method that supports researchers to explore perspectives among participants (Nowell et al., 2017). After obtaining the participants' permission, the interviews were audio-recorded, transcribed verbatim, and sorted based on the questions asked to the participants. Furthermore, the transcripts were exclusively utilized for the thematic analysis and did not disclose the identities of the participating students. The thematic analysis was performed using the data acquired from the interview transcripts to construct groupings and patterns. Furthermore, throughout the analysis, keywords were converted into codes by highlighting significant sentences in the transcripts. These specific keywords were extracted based on an inductive open coding approach and shortened to codes. The retrieved codes served as the foundation for bundling and arranging the data into clusters, as well as thematically assessing the material (Terry et al., 2017). This approach was continued until the results were agreed upon by the principal researcher and research assistant. Finally, conclusions were reached based on the identified categories and patterns of perceived usefulness of the in-house training program.

### 2.5 Confidentiality and ethics

The particular objective and scope of the research were explained to interview participants, and written consent was acquired prior to the interviews. The permission form was created in accordance with the policies of the South East Sweden's Ethical Advisory Board (2021). The students were informed that they may resign from the research at any moment and that their data might be erased at any time. Furthermore, the respondents were informed that their participation in the study had no influence on their academic achievement. Furthermore, confidentiality was extended to all 25 respondents, the names of whom were only known to the researchers participating. Finally, as a token of gratitude for their time and thoughtfulness, the participants were given gift cards at the end of the interviews.

### 2.6 Environment

The study was conducted with participants at the Prince of Songkla University. As a result of the ongoing pandemic, many businesses in the hospitality and tourism industry continue to struggle with their business continuity (Fuchs, 2021) in absence of international tourism (Romagosa, 2020). Consequently, fewer internship opportunities were available or students were opposed to last-minute cancellations due to the worsening of the global pandemic in Thailand. Therefore, the management of the Faculty of Hospitality and Tourism, Prince of Songkla University developed and implemented an in-house training program for their undergraduate students. A total of 76 s and fourth-year undergraduate students opted for this alternative and subsequently registered for the four-month in-house training starting in early January 2022. Irrespective of their major, the students could freely pick between an Entrepreneur Prep Up program and a Tour Guide Prep Up program. There were no additional costs for the students to participate in the program, which consists of theoretical classes, lectures by industry professionals, workshops led by university lecturers, project-based learning, field trips, and learning assignments.

## 3 Results

The following subsections report on the four themes that emerged during the thematic analysis of the empirical data. Namely, these themes are (1) motivators and factors to complete the in-house training program, (2) competence development during the in-house training program, (3) practical emphasis with a theoretical application, and (4) linkage with industry professionals.

### 3.1 Motivators and factors to complete the in-house training program

The students stated that they were given three choices by the internship administration of the university. First, they could conduct a regular industry placement, which appeared to be the preferred choice by the majority of students. Second, they could attend the newly emerged in-house training program as an alternative to the industry placement. Third, students could apply for academic leave for the duration of one semester without paying full tuition fees. Furthermore, the opening question of the interview asked the participants to describe their motivation to join the in-house training program instead of attending an industry placement. The students reported that as a result of the pandemic, many internship sites canceled the industry placement for the participating students (*n* = 17). However, this is not the only factor that the students reported as a motivator to join the alternative in-house training. Other factors that the students reported were avoidance of high-risk areas (*n* = 4), convenience (*n* = 3), and personal expenditure (*n* = 1) as summarized in Table 3.

**Table 3:** Summary of reported motivators to join the in-house training program.

Frequency	Motivator/factors	Description
*n* = 17	Cancellation of industry placement	Internship site cancelled the industry placement
*n* = 4	Avoidance of high-risk areas	Avoiding human contact with crowds in high-risk areas
*n* = 3	Convenience	Ease to complete the internship in-house opposed to externally
*n* = 1	Personal expenditure	Costs associated to conduct the internship, e.g. staff uniform, meals, transportation

Moreover, the students whose internship was canceled also implied during the interview that they attempted to find a new industry placement. However, the lack of available options or deadline to report their internship place with the administration of the university prevented them from doing so. Nonetheless, no participating student considered taking academic leave to attend an industry placement at a later time in their studies. The sole response from the students was “to avoid falling behind on their studies” as the primary reason.

### 3.2 Competence development during the in-house training program

In the context of this study, competence is defined as the overarching term that includes skills, knowledge, and attitude. The students were specifically asked about their expectations towards the in-house training program, as well as to self-report their perceived competence development. All of the participating students (*n* = 25) were able to recognize that their competencies improved through the attendance of the program. In particular, their knowledge related to their academic majors improved significantly as demonstrated by the enclosed verbatim that is representative of the vast majority of students.

“I gained a lot of new knowledge during the training. But no in the classroom. Most of the knowledge came from the guest speakers that visited us, or when we go on a field trip. But also, during the projects that my team and I worked on” (P15)

Moreover, the students reported that their presentation skills (*n* = 22), communication skills (*n* = 19), and collaborative working skills (*n* = 14) improved through the in-house program, which can be accredited to the workshop activities and variety of learning assignments.

“We had to do a lot of presentations. A lot. I didn’t like it at first, but in the end, I could see that I really benefited from it and became also more confident to speak in front of others” (P8)

“I would say that teamwork and presenting are the two skills that improved the most. I am actually quite thankful for it because I wouldn’t volunteer to present normally, but this time, we all had to present our projects” (P3)

Nevertheless, most students (except for two students) informed that the lack of a real-life situation impacted their motivation and engagement negatively throughout the program. The interviewees commented that they felt insufficiently challenged (during the in-house training) to improve their problem-solving skills (*n* = 13), creative-thinking skills (*n* = 13), as well as, critical-thinking skills (*n* = 11) as attested by the statements from the interviewees P2 and P18.

“I wish that I would be put in the real-life situation. It’s just not the same when I have to solve a problem within my group, or with customers at the hotel. Therefore, I think, I should improve more” (P2)

“I wasn’t really pushed to the limit to improve skills to solve difficult situations or think critically because it wasn’t the real. I think that I would react differently in a situation with real customers” (P18)

Overall, it can be noted that all students perceived that their competencies improved through the training. However, the elderly students (i.e. fourth-year) were more critical in comparison to their young peers (i.e. second-year). The majority of elderly students reported that “their competencies improved, but would have improved more [with an internship]”.

### 3.3 Practical emphasis with a theoretical application

There was a significant difference in the reporting when comparing the results from the second-year students (who would not have a point of comparison other than their lecture-based classes) with the results from the fourth-year students. The latter would have been exposed to an industry placement two years prior to conducting the study, and henceforth, have a different point of comparison in contrast with their younger peers. The fourth-year students would unanimously report that the in-house training was, to a very large extent, theory-based with little to no new knowledge to be gained.

“At times, it was so boring. We would just sit in the classroom and after a while, I get demotivated and start to chitchat with my friend. I mean, we just have to listen, sometimes even for hours, and the information feels like a summary of what we already know. Is it even training or just another lecture?” (P24)

These comments are in direct contrast with the comments made by the second-year students who concurred to the theoretical basis (i.e. lecture-based learning), but also reported that the overall application of the in-house program was more interactive compared with their regular coursework.

It was like a normal class, but with more activities and more presentations. Normally, when we don’t have the in-house training, we would join a lecture for 3 h, but during the in-house training, we had more group work and guest lecturers visiting us. It was more interesting than a normal class, but I am not sure if I can compare it with a real internship” (P17).

Similarly, most students (irrespective of their year of study or academic major) agreed that the content of the lectures was not tailored to their needs. In particular, the second-year students remarked that the theoretical content was “overwhelming”, “rushed” and “not well explained”, wherein their fourth-year peers would state that the theoretical content was merely a summary of “what we know already from previous years”. Similarly, the enclosed verbatims are representative of the sentiment shared by most of the students.

“It’s the same information from my last two years at the university. I didn’t learn a lot of new information in the class. But I received new knowledge in the practical parts when we were in the laboratory [to cook dishes]” (P5)

“Yes, it wasn’t really new. At least not for me and my friends, but I think the younger students might learn something that is helpful for their future” (P11)

Furthermore, the students were asked about their collaboration with peers from a different year or different major, wherein the younger students expressed interest to collaborate with their elderly peers, however, felt often intimidated to approach them during the workshops and teamwork exercises.

“I really wanted to work with my seniors, but they already had their group for the project and I was too shy to ask them to join. It was easier to stay with my friends” (P2)

“It’s a missed opportunity because I think we can learn from the experience of our seniors, but it was just easier to stay in groups with people that we know. The teachers didn’t ask us to mix the groups” (P9)

Generally, all students agreed that the field trips (to different tourist sites or visiting successful businesses in the area) were perceived as one of the best aspects of their in-house training program. Most students wished for fewer theoretical classes and more field trips “to experience real-life situations, and not talk about situations from the book”.

### 3.4 Linkage with industry professionals

A strength of the in-house training program was the reported links with the industry. When the students were asked about the best aspects of the program, 23 out of 25 would respond that the guest lectures or visitors from the company were most appreciated.

“They shared experiences from their daily work life and it felt quite inspiring to listen to them” (P3)

“I would say that the best aspect was definitely the guest speakers. They gave us a really good idea about what life after university would be like” (P6)

“It was great to hear it from someone that is actually doing the job. I would prefer less theory lectures and more guest lecturers” (P12)

Moreover, a few students noted that lack of networking opportunities was seen as a shortcoming of the program (see verbatim below), wherein some of the elderly students voiced that inviting business representatives helped to overcome this.

“Of course, an internship is to develop my skills and get experience. Also, it helps me to check what I really want to do after I graduate. But I also worry that I don’t get enough connections with businesses. In Thailand, many jobs depend on whom you know” (P1)

“I am regretful for not getting the opportunity to network outside the university. It is quite important to make connections with other staff at the hotel [or airport] because it’s easier to apply for a job there later on” (P13)

Furthermore, about half of the participating students reported that the representatives from hospitality and tourism businesses gave more meaningful and motivating lectures as they related to “real-life experiences” rather than “studying from the textbook”.

## 4 Discussion

Internships are quickly becoming a required component of an entry-level job applicant's portfolio (McHugh, 2017). However, we are just now beginning to grasp how internships differ in design and quality, and how these variations might affect the effectiveness of the internship experience (Qu et al., 2021). The study contributes by exploring how undergraduate students experience the perceived usefulness of their internship experience amid COVID-19. According to a recent study by Chalapati and Chalapati (2020), Thailand is being confronted with a severe shortage of skilled workforce, not limited to but including an employee shortage in the hospitality and tourism industry (p. 68). This prevents the Thai economy from achieving substantial economic progress (Chalapati and Chalapati, 2020) as well as further adds fuels the risk of losing its global competitiveness standing compared with other tourism destinations in the world (Dogru et al., 2021). Employees in hotels, airports, restaurants, or tour companies need to be competent, qualified, as well as well-trained.

The educators and institutions have a key role to educate and prepare the students for the challenges in the workplace (Pusiran et al., 2020). Therefore, it is important for vocational institutes and higher education institutions to produce high-quality graduates, which includes the application of practical training and workplace experiences (Singh and Sharma, 2021). The broader sentiment of most students and the associated empirical findings do not augur well for the in-house training program as a replacement for an industry placement. Similarly, all fourth-year students and the majority of second-year students claimed that the lack of real work experience in an unrealistic setting as the primary drawback of the in-house program. Nevertheless, upon closer examination, it transpired that a variety of significant findings and recommendations emerged.

One of the main implications arising from this study relates to the need of developing a purposeful framework for the in-house training instead of merely creating a mechanism that allows students to graduate within the stipulated period of the program as similarly reported by Park and Jones (2021). The gap between second-year and fourth-year students has the potential to create value if applied correctly (Storey et al., 2021). The students voiced their concern that the information was overwhelming for second-year students while demotivating for fourth-year students. A possible way to capitalize on the knowledge gap could be peer-lecturing (peer-to-peer), wherein older, more experienced students teach their younger, less experienced peers. It creates a more meaningful experience for both groups of students, and facilitates active learning, wherein the lecturer moves into the role of a facilitator (Lowton-Smith et al., 2019). Comparably, it would also stimulate the group of students that claimed a lack of active involvement as a shortcoming of the in-house program as similarly reported by Pusiran et al. (2020) in a related study.

De Vos et al. (2011) note that competency development during undergraduate studies, in particular during their internship period, plays a significant role in the students’ employability upon graduation. Moreover, competence development is necessary to equip students with the needed knowledge, skills, and attitudes to successfully navigate their transition from university to the workplace (Plewa et al., 2015; Spanjaard et al., 2018). The participants of the study agreed that their verbal communication skills and presentation skills significantly improved during the in-house training. Moreover, a notable boost in confidence was reported as one of the positive side effects of attending the training. However, many of the participants reported insufficient development of their critical-thinking, creative-thinking, and problem-solving skills. While the thought emerges that the nature of the in-house training cannot sufficiently achieve the same competency developmental outcomes as an industry placement, it should also be noted that a variety of tools can supplement and improve the learning experience (Ye and Law, 2021). ChanLin and Hung (2015) are reporting that students reacted positively to the learning and reflective processes embedded in journal writing as part of their online internship. These tools have the potential to improve critical-thinking and creative-thinking skills (ChanLin and Hung, 2015), which were reported as shortcomings during the in-house training program. Another avenue to compensate for potential shortcomings could be the addition of extracurricular activities or elective course to foster the development of soft competencies (Xu et al., 2022).

As far as the educational provider is concerned, a reoccurring comment made by the vast majority of participants was the successful addition of industry professionals into the lectures and workshops. In particular, the participants of the program enjoyed and appreciated learning about real-life experiences from someone that is doing the job. These findings are on par with Maini et al. (2021) who state that interaction with the industry has a potential role in the successful implementation of alternative internship program. While the integration of industry professionals as part of the in-house program provided valuable insights for the participants, it is recommended to expand and foster the relationship between educational providers and the industry. As noted by Remington and Pellicano (2019) that work placements appear to be an important step to promote employment outcomes (p. 516). This can be achieved by developing a framework for the in-house training programs that allow for comments and inputs by industry professionals and their workplaces. It will create a closer bond between the industry and university, as well as create added value for the participants of the in-house training program.

This research contributes to both academics and the tourism industry. This study generated a broad list of themes that impacted the internship experiences and empirically evaluated them among Thai undergraduate students. The study has implications for both tourism and hospitality educators and industry practitioners. Internship programs connect tourism and hospitality education to industry experience and there play an important role in addressing the identified challenges. To expand internship possibilities, universities should construct and strengthen workplace relationship building, management, and training processes. In particular, integrating industry professionals in the planning, implementation, and execution of in-house training programs improves the perceived usefulness of the program. Further, it provides a networking link for students to ease into an entry-level position upon graduation. Collaboration between educational institutions and industry might also benefit from better understanding each other's aims and coordinating student experiences to enhance learning and beneficial outcomes.

There are three major lessons for institutions in higher education that wish to implement or improve an in-house training program. First, a linkage with the industry is considered a must-have for implementing a practical training program. Second, competency development should be at the core of the program. Students can improve their knowledge through coursework (Vo et al., 2021), therefore, the training program should emphasize improving cognitive skills as relatedly suggested by Anjum (2020) in a different geographical context Third, the program is better suited for younger, less experienced students as a possible prerequisite for actual industry placements. This implication is further supported by Zehr and Korte (2020) who note that it is essential for younger undergraduate students to have positive experiences with their first work-life connection, wherein the development of cognitive skills and networking opportunities are increasingly important for elderly students toward the end of their degree program.

## 5 Conclusion

The study aimed to explore how undergraduate students in tourism and hospitality education perceive the usefulness of their in-house training in absence of an industry placement. To sum up, it can be noted that in-house training programs are not a sustainable substitute that can be considered a permanent alternative for an industry placement in the current form. However, the empirical findings of the study and derived implications offer suggestions on how the in-house program can be transferred into a more sustainable solution. Limitations always offer an opportunity for future research and the results of the study should be evaluated in their appropriate context. Therefore, the reader is advised that the results of this study are not directly transferrable into the context of other academic majors or different geographical settings. However, the practical recommendations offer novel insights for educators and educational institutions to improve the educational quality of their programs, and therefore, enhance the quality of graduates the faculty produces. Moreover, the findings stimulate areas of future work for educational researchers. For example, it is yet unknown how in-house training programs affect the employability of the students or their core competency development during their undergraduate studies.

## Institutional approval

This study was approved by the Research Committee of the Faculty of Hospitality and Tourism, Prince of Songkla University on 28. February 2022 (approval no. FHT65000004).

## Informed consent

All participants provided written informed consent prior to enrolment and participation in the study.

## Declarations

### Author contribution statement

Kevin Fuchs: Conceived and designed the experiments; Performed the experiments; Analyzed and interpreted the data; Contributed reagents, materials, analysis tools or data; Wrote the paper.

### Funding statement

This work was supported by the Faculty of Hospitality and Tourism, Prince of Songkla University under the Fast Track Data Collection Grant.

### Data availability statement

Data will be made available on request.

### Declaration of interest’s statement

The author declares no competing interests.

### Additional information

No additional information is available for this paper.
